# Evaluation of wound healing and anti-inflammatory activity of the rhizomes of *Rumex abyssinicus* J. (Polygonaceae) in mice

**DOI:** 10.1186/s12906-015-0878-y

**Published:** 2015-09-30

**Authors:** Eshetu Mulisa, Kaleab Asres, Ephrem Engidawork

**Affiliations:** Department of Pharmacology and Clinical Pharmacy, School of Pharmacy, College of Health Sciences, Addis Ababa University, P.O.Box 1176, Addis Ababa, Ethiopia; Department of Pharmaceutical Chemistry and Pharmacognosy, School of Pharmacy, College of Health Sciences, Addis Ababa University, Addis Ababa, Ethiopia

**Keywords:** *Rumex abyssinicus*, Rhizome, Excision model, Incision model, Anti-inflammation, Wound healing

## Abstract

**Background:**

*Rumex abyssinicus* Jacq (Polygonaceae) is widely used in Ethiopia for treatment of wound and other diseases. Although reports are available in the literature on some of the claimed activities, nothing has so far been reported about the wound healing activity of *R. abyssinicus.* Thus, this work was initiated to investigate the wound healing and anti-inflammatory activities of 80 % methanol extract of the rhizomes of *R. abyssinicus* in mice.

**Methods:**

Following extraction of the rhizomes of the plant with 80 % methanol, the extract was formulated as ointment (5 % & 10 % w/w) with simple ointment base B.P. The ointment was then evaluated for wound healing activity using excision and incision wound models. Parameters, including wound contraction, epithelization time and hydroxyproline content were determined using the excision model, whereas tensile strength was measured from the incision model. In parallel, anti-inflammatory activity of the rhizome was evaluated with carrageenan induced hind paw edema model by dissolving the 80 % methanol extract in 1 % carboxyl methyl cellulose and administering orally in various doses (250, 500 and 750 mg/kg).

**Results:**

Wound treated with 5 % and 10 % (w/w) hydroalcoholic extract ointment exhibited significant wound healing activity in both models, as evidenced by increased wound contraction, shorter epithelization time, higher tissue breaking strength and increased hydroxyproline content. The hydroalcoholic extract also produced dose-related significant reduction (*p* < 0.05–0.001) of inflammation.

**Conclusions:**

The results of this study demonstrated that the hydroalcoholic extract of the rhizomes of *R. abyssinicus* facilitated wound healing at least in part via its anti-inflammatory activity, supporting its traditional claim as a wound healing agent.

## Background

Wound may be described in different ways but the most common definition is “loss or breaking of cellular and anatomic or functional continuity of living tissues” [1, 2], and it can be broadly categorized as acute or chronic wound [3, 4]. The healing of wound is a complex process. At the time of injury, capillary will be damaged and blood clot is formed. This is followed by an early stage of inflammation. During the early phase of inflammation, cells like neutrophils and monocytes clean bacteria and necrotic tissue through phagocytosis as well as release of enzymes and toxic oxygen products. This is followed by migration of macrophage to wound area, which marks the transition from early to late phase of inflammation. The proliferative phase, which follows, is characterized by granulation and tissue proliferation formed mainly by fibroblast and the angiogenesis process. Finally, reformulation and improvement in the components of the collagen fiber that increases the tensile strength will occur during the remodeling stage [3]. Thus, alterations in the inflammatory phase will impact the overall integrity of the healing wound.

Globally, wounds are a main cause of disability and lost productivity. Apart from the incidence, chronic wounds are known for causing different health problems. A study done in North European countries indicated that foot ulcers cause up to 85 % of amputations [5, 6]. In sub-Saharan African and South Asian countries, 1 % to 2 % of the population faces chronic wound at least once in their lifetime [7, 8]. More than 1.25 million people sustain burns and approximately 5 million suffer from non-healing wounds each year in the US [9]. The cost of wound care is substantial. Chronic wounds cost European countries 1 % to 2 % of their annual health care budget and $1 billion/year in the US [10]. This cost is unthinkable for most people in developing countries suffering from infected wounds [7]. Indirect costs such as psychosocial damage and poor quality of life are also major concerns [11].

Majority of the population in both developing and some developed countries use traditional medicine for their primary health care [12, 13]. In developing countries, like Ethiopia, management of skin diseases and dermatological disorders such as cuts, wounds, and burns has relied on medicinal plants for a long period of time. Herbs are known to make the wounded area moist, which is very essential for healing [14, 15]. Nowadays, discovery is shifted towards wound healing promotion in order to reduce hospitalization cost and serious complications [16]. Several lines of evidence have shown the benefits of a wide variety of plants in their wound healing capacity [2, 17]. Thus, plant products are considered to be the best and cost-effective substitutes for wound treatment.

*Rumex abyssinicus* Jacq (Family; Polygonaceae) is the most common traditional medicinal plant in the highlands of tropical Africa and distributed throughout North Africa and Ethiopia. In the Ethiopian traditional medicine, the rhizomes of *R. abyssinicus* (“Mekmeko” in Amharic) are used to treat malaria, gonorrhea, poisoning, hepatitis, constipation, sciatic neuralgia, hypertension, migraine, rheumatism, breast cancer, stomach distention, earache, liver diseases, hemorrhoids, typhus, rabies and wound [18–22].

Previous reports indicated that 80 % methanol extract of the rhizomes of *R. abyssinicus* possessed antimicrobial and *in vitro* anti-inflammatory activities [21], diuretic and analgesic [22], and antimalarial [23] activities. To date, however, no scientific report could be found in the literature concerning the wound healing activity of the plant, although there is an ample ethnobotanical claim for this property. This study was therefore conducted to provide scientific evidence for the folklore use of the plant in wound healing processes.

## Methods

### Plant material

The fresh rhizomes of *R. abyssinicus* was collected from Menagesha forest, located 40 km west of Addis Ababa, Ethiopia. The plant was identified by a taxonomist and a voucher specimen (E001/10) was deposited at the National Herbarium, College of Natural and Computational Sciences, Addis Ababa University (AAU) for future reference.

### Experimental animals

Healthy, adult Swiss albino mice of either sex (25–30 g, and 6–8 weeks of age) purchased from the animal house of the Ethiopian Public Health Institute and adult Wistar rats of both sexes (200–300 g, aged 3–4 months) obtained from the animal house of the School of Pharmacy, AAU, were used for the study. The animals were housed in cages under standard conditions (25 ± 2 °C, 55 ± 5 % relative humidity, and 12 h light and dark cycles) and provided with pellet diet and water *ad libitum*. The study protocol was approved by the Ethics committee of the School of Pharmacy, AAU. Animal handling and care was carried out throughout the experiment following international laboratory animal use and care guidelines [24].

### Plant extraction

Rhizomes of *R. abyssinicus* were sliced to smaller pieces and dried for three weeks under shade. The dried rhizomes were then grinded to coarse powder. Five hundred gram of the powder was then macerated with 80 % methanol for three days in a conical flask with occasional stirring and shaking. The extract was then filtered (Whatman No. 1) and the residue was re-macerated twice to obtain maximum yield. The combined filtrate was then evaporated in a ventilated oven at 40 °C until dried. The resulting dry extract was weighed and provided a percentage yield of 16.2 % (w/w). The dried extract was stored in a refrigerator for the preparation of topical formulation (ointment).

### Ointment formulation

Simple ointment of the 80 % methanol extract was prepared following the formula (Table 1) described in the British Pharmacopoeia [25]. Three ointment preparations (each 200 g), with (5 % and 10 % w/w) and without (simple ointment only and served as a control) the extract were formulated using the reduced formula from the master formula (Table 1). All ingredients of the ointment base were mixed and heated gently, with stirring until homogenous and then stirred until cooled. For preparing medicated ointment, 10 g and 20 g of the 80 % methanol extract were mixed with 190 g and 180 g of the ointment base, respectively, by levigation on the surface of the ointment slab to make ointment of uniform consistency and smooth texture [26]. In preparing the control ointment, 200 g of the base was taken and treated in the same manner to formulate ointment without an active ingredient.

**Table 1:** Formula used for preparation of the ointment

Ingredients	MF	RF
Wool fat	50 g	10 g
Hard paraffin	50 g	10 g
White soft paraffin	850 g	170 g
Cetostearyl alcohol	50 g	10 g
	1000 g	200 g

MF, Master formula; RF, reduced formula

### Acute dermal toxicity

For dermal toxicity, a total of 10 (5 female and 5 male) rats were used. Animals showing normal skin texture were housed individually in a cage and acclimatized to the laboratory condition for five days prior to the test. Following acclimation, around 10 % of the body surface area fur was shaved 24 h before the study from the dorsal area of the trunk of the test animals. A limit test dose of 2000 mg/kg of the 10 % formulation was applied uniformly over the shaved area for 24 h. At the end of the exposure period, residual test substance was removed and the animals were observed for development of any adverse skin reactions daily for 14 days.

### Grouping and dosing of animals

For excision model, four groups of mice, each containing six animals were used. The first group was treated with simple ointment, and served as a negative control. The second and third groups were treated with 5 % and 10 % of the extract ointment, respectively. The fourth group was treated with nitrofurazone (0.2 %) and served as a positive control. For incision model, five groups of mice, containing six mice per group were used. The animals of Group I-IV were treated in a similar fashion with excision wound model, but animals in Group V were not treated with any agents and served as untreated controls.

For assessment of anti-inflammatory activity, five groups of mice containing six animal per group were used. Group I was treated with 1 % carboxyl methyl cellulose (CMC) and served as a negative control. Groups II-IV were treated with 250 mg/kg, 500 mg/kg, and 750 mg/kg of the extract. Group V was treated with indomethacin (10 mg/kg). Dose levels were chosen based on acute oral toxicity results described elsewhere [22] and dose calculation was based on Deshmukh et al. [27], with slight modification. All administrations were performed orally, with a maximum volume of 10 ml/kg. The extract and the standard drug were dissolved in 1 % CMC to obtain an oral suspension.

### Excision wound model

On wounding day, animals were anesthetized using subcutaneous injection of ketamine (1 ml/kg) and the back hair of the animals was depilated by shaving. About 300 mm^2^ circular area was then marked and the full thickness of the marked area was carefully excised by using sharp sterilized scissors. After 24 h of wound creation, the ointments were applied gently once daily, according to the respective grouping as described under grouping and dosing section, to cover the wounded area until complete healing was achieved. Wound contraction, epithelization period and hydroxyproline content were monitored. Wound contraction was measured as percent contraction every 2 days until complete wound closure was achieved [12].

#### Measurement of wound contraction

The wound healing progress was evaluated by measuring wound areas using a transparency sheet and a permanent marker. The evaluated surface area was used to calculate the percentage of wound contraction, taking initial size of the wound (300 mm^2^) as 100 % [28] as shown below:

$$ \%\ \mathrm{Wound}\ \mathrm{contraction}=\frac{\mathrm{Initial}\ \mathrm{wound}\ \mathrm{size}\ \hbox{-}\ \mathrm{specific}\ \mathrm{day}\ \mathrm{wound}\ \mathrm{size}}{\mathrm{Initial}\ \mathrm{wound}\ \mathrm{size}\ }X100 $$

#### Epithelization time measurement

The period of epithelization was calculated as the number of days required for falling off of the dead tissue remnants without any residual raw wound [29].

#### Estimation of hydroxyproline content

**Fig. 1:**
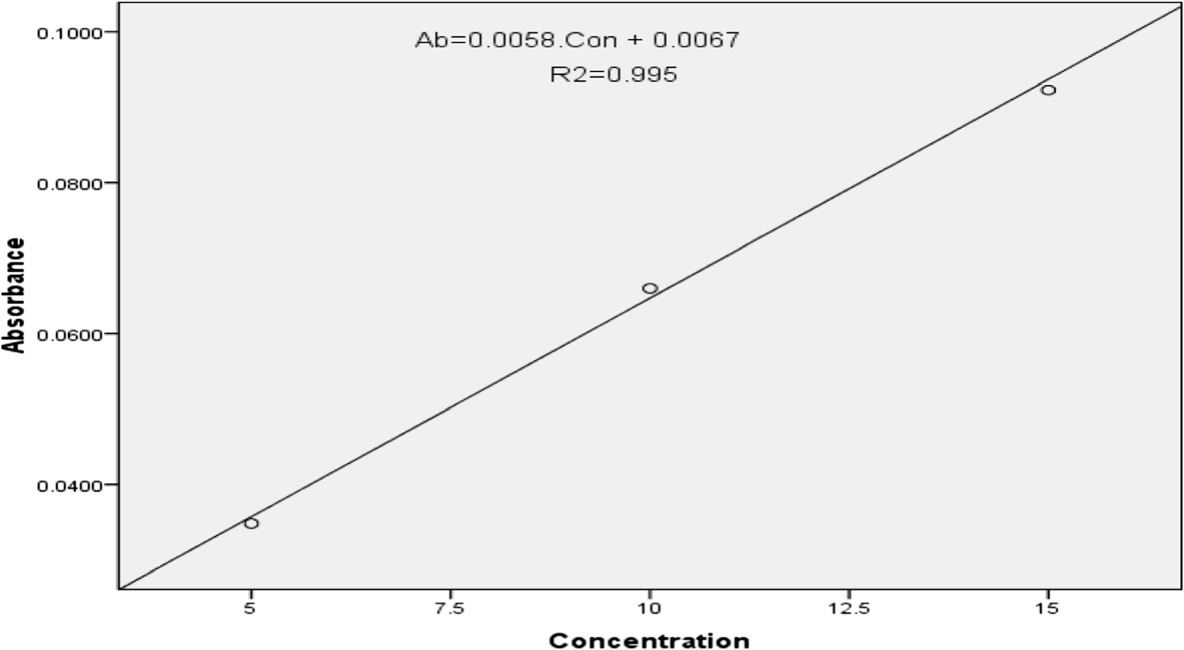
Calibration curve for estimating hydroxyproline concentration: tissue obtained from excision wound was hydrolyzed in 6 N HCl for 24 h at 110 °C in sealed glass tubes. The hydrolysate was neutralized to pH 7. One ml of the supernatant solution was taken from each tissue hydrolysate and the absorbance was read

Following treatment with the formulations for 10 days of the circular wound created in the excision model, each animal from the respective group was killed on the 11^th^ day using a high dose of anesthesia. The wound tissue was then excised and its weight was recorded and placed in 10 % formalin and stored in a refrigerator. On the day of the experiment, the tissue was dried in an oven at 60 °C for 12 h and the dry weight was again noted. The tissue was then hydrolyzed with 6 N HCl for 24 h at 110 °C in sealed glass tubes. The hydrolysate was neutralized to pH 7 [30]. One ml of the supernatant solution was taken from each of the acid hydrolysate and treated in the same way to the standard hydroxyproline [31] and absorbance was determined at a wave length of 572 nm in 1 cm cell. The hydroxyproline content in each of sample solution was calculated using the equation which was obtained from the calibration curve (Fig. 1).

### Incision wound model

**Fig. 2:**
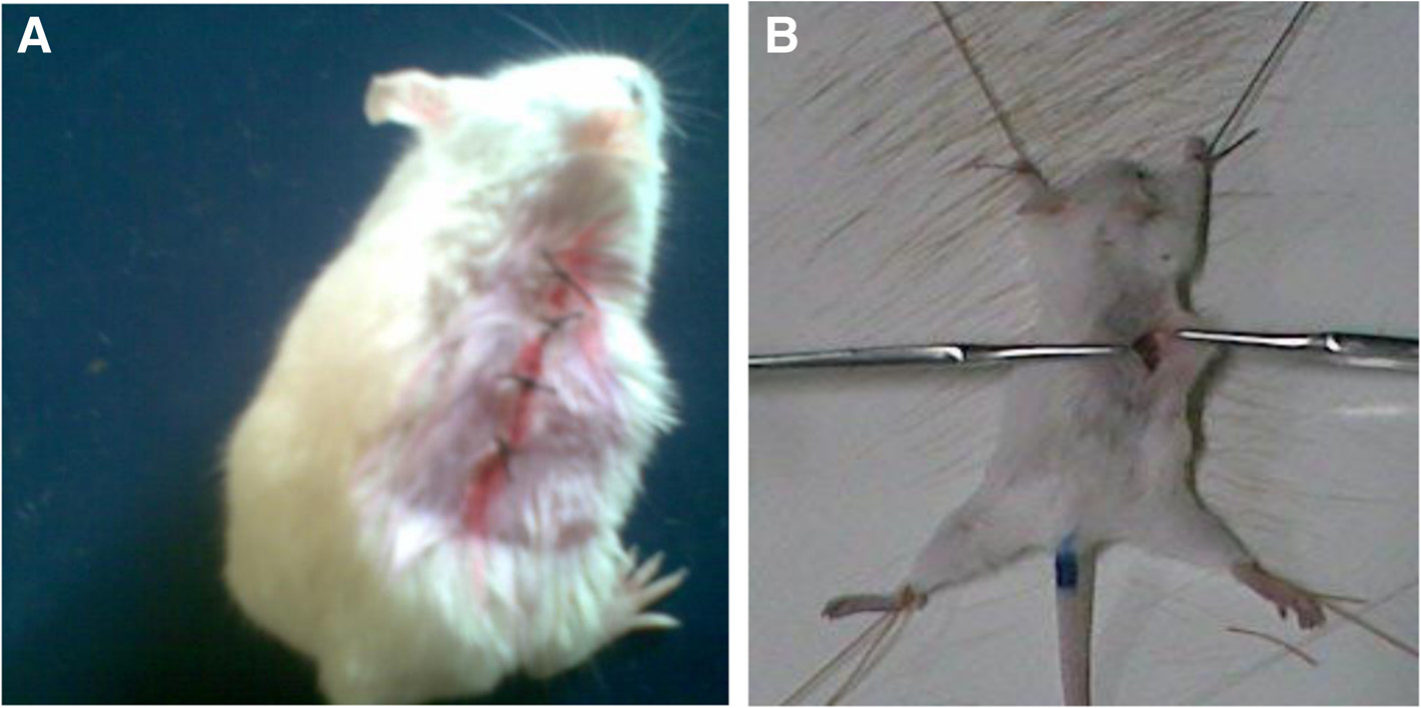
Incision wound creation (**a**) and tensile strength measurement (**b**): The animals were anesthetized and then positioned on a tray between two metal stands. To both stands, forceps of equal size were suspended with strings, one of which passing on a small wheel with the stands. Constantly flowing water was then allowed to enter into the plastic bottle, which was suspended with the string paasing on the wheel until the wound breaks. Wound was created at day 0 and treatment was started 24 h after wound creation (1^st^ day) for nine days. The sutures were removed on day 8 post-incision and tensile strength was measured on the 10^th^ post-wounding day using continuous water flow technique

On wounding day, animals were anesthetized in the same manner described for excision wound model. The dorsal fur of each mouse was then shaved and a 3 cm long longitudinal paravertebral incision was made through the skin and subcutaneous tissue. The parted skin was then sutured 1 cm apart using a surgical thread (no. 000) and curved needle (no. 11). The continuous thread on both wound edges was tightened for good closure of the wounds (Fig. 2a).

After 24 h of wound creation (on 1^st^ day), animals were treated as described under grouping and dosing section, with topical formulation of vehicle, extract or standard daily for nine days, leaving out the last group which did not receive any of the interventions. The sutures were removed on day 8 post-incision and tensile strength was measured on the 10^th^ post-wounding day using continuous water flow technique (Fig. 2b) [32] according to the formulas shown below [33].

$$ \mathrm{Percent}\ \mathrm{T}\mathrm{ensile}\ \mathrm{strength}\ \left(\mathrm{T}\mathrm{S}\right)\ \mathrm{of}\ \mathrm{extract} = \frac{\mathrm{TS}\ \mathrm{extract}\ \hbox{-}\ \mathrm{T}\mathrm{S}\ \mathrm{so}\ }{\mathrm{TS}\ \mathrm{so}\ }X100 $$

$$ \mathrm{Percent}\ \mathrm{T}\mathrm{ensile}\ \mathrm{strength}\kern0.5em \mathrm{of}\ \mathrm{reference} = \frac{\mathrm{TS}\ \mathrm{reference}\ \hbox{-}\ \mathrm{T}\mathrm{S}\ \mathrm{so}\ }{\mathrm{TS}\ \mathrm{so}\ }X100 $$

$$ \mathrm{Percent}\mathrm{T}\mathrm{ensile}\mathrm{s}\mathrm{trength}\kern0.5em \mathrm{o}\mathrm{f}\mathrm{s}.\mathrm{o}=\frac{\mathrm{TSso}\hbox{-} \mathrm{TSlu}}{\mathrm{TSlu}}X100 $$

Where so is simple ointment, and lu is left untreated.

### Anti-inflammatory activity

Anti-inflammatory activity of *R. abyssinicus* was determined using mouse paw edema model. Following overnight fasting with free access to water, the basal volume of the right hind paw of each mouse was determined before administration of any drug using plethysmometer (Ugo Basile, Italy) [34]. After determination of the basal volume, the animals were divided into five groups such that the mean volume of the different groups were similar. The mice were then treated as described under grouping and dosing section 1 h before carrageenan injection. Paw swelling was induced by subplantar injection of 0.05 ml of a solution of 1 % carrageenan in 0.9 % saline (w/v) into the right hind paw. The inflammation was quantified by measuring the volume displaced by the paw 1, 2, 3 and 4 h after carrageenan injection. Results were expressed as the paw volume (ml) variation with respect to the basal values. The percentage inhibition of edema for each group was calculated using the following formula [35]:

$$ \mathrm{Percentage}\kern0.5em \mathrm{inhibition}\ \mathrm{of}\ \mathrm{edema} = \frac{\mathrm{Co}\ \hbox{-}\ \mathrm{C}\mathrm{t}}{\mathrm{Co}}X100 $$

Where *C*_o_ is the average inflammation (hind paw edema) of the control group at a given time; and *C*_t_ is the average inflammation of the plant extract or indomethacin treated mice at the same time.

### Statistical analysis

Raw data obtained from both wound and mouse paw edema models are expressed as mean ± SEM. The data were analyzed using SPSS version 17.0 and differences among groups were compared with one-way ANOVA followed by Post Hoc Tukey test. The data were considered significant at *p* < 0.05.

## Results

### Acute dermal toxicity

**Fig. 3:**
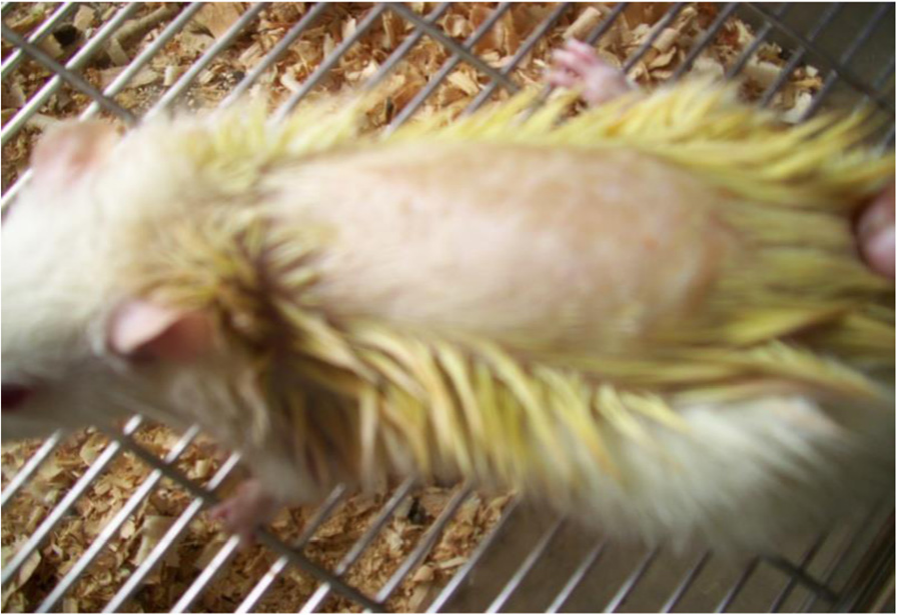
Photograph of skin 24 h after application of 10 % ointment of 80 % methanol extract of the rhizome of *Rumex abyssinicus* for acute dermal toxicity study: a limit dose of 2000 mg/kg was applied topically to assess dermal toxicity and no sign of inflammation was observed

Maximum concentration of the ointment (10 % (w/w)) administered at a limit dose of 2000 mg/kg was found to be safe. After 24 h of application of the ointment, the site did not show any sign of inflammation (Fig. 3). There were also no overt signs and symptoms observed when the animals were monitored for 48 h. Moreover, no signs of toxicity as well as no mortality were noted during the 14 days cage side observation.

### Effect on excision wounds

#### Wound contraction

**Fig. 4:**
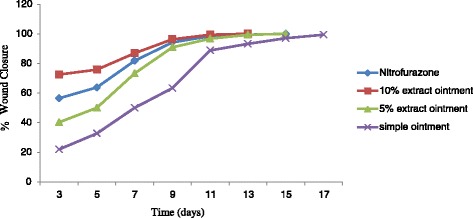
Effect of 80 % methanol extract of *Rumex abyssinicus* rhizomes on the percentage wound closure of the excision wound model: *n* = 6 animals in each group; Values are expressed as mean ± SEM; SO, simple ointment base; HEO, hydroalcoholic extract ointment; NFO, nitrofurazone ointment. Percent wound closure increased significantly with the extract across time compared to controls. Maximum closure was achieved on day 11, which was 13 and 15 from the standard and vehicle, respectively

Topical application of both 5 % and 10 % of the hydroalcoholic extract ointments in excision wound model significantly increased wound contraction rate compared to controls, with variable onset of action (Table 2 & Fig. 4). Whilst 5 % ointment produced a significant contraction on the 7^th^ day (*p* < 0.01), 10 % produced on the 3^rd^ day (*p* < 0.001). In addition, the contraction produced by the 10 % ointment was significantly greater (*p* < 0.01) than that of the 5 % on the 3^rd^ and 5^th^ day. No apparent difference was, however, observed thereafter between the two concentrations until the end of the observation period (Fig. 3). The 10 % ointment also appeared to provide better wound contraction than the reference compound until day 13, although the difference failed to reach statistical significance.

**Table 2:** Effect of topical application of the ointment formulated from 80 % methanol extract of the rhizomes of *Rumex abyssinicus* on wound contraction of excision wound model in mice

Group	Wound area (mm^2^) on post-wounding days						
	3	5	7	9	11	13	15
SO	234.2 ± 11.8	202.0 ± 10.6	159.1 ± 13.0	110.0 ± 8.5	33.5 ± 5	20.5 ± 2.7	9.2 ± 3.0
5 % HEO	179.3 ± 23.3	150 ± 15.0	80.2 ± 21.7^a2^	27.3 ± 11.4^a2^	10 ± 6^a2^	2 ± 1.4^a3^	0^a2^
10 % HEO	82.4 ± 8.1 ^a3,c2^	72.67 ± 9.2^a3,c2^	39.1 ± 8.1^a3^	11.0 ± 3.0^a3^	1.8 ± .5^a3^	0^a3^	-
NFO	130.7 ± 17.0^a2^	108.5 ± 17.4^a3^	54.5 ± 10.7^a3^	17.2 ± 4.8^a3^	4.8 ± 2^a3^	1.67 ± 1.3^a3^	0^a2^

*n* = 6 animals in each group; Values are expressed as mean ± SEM and analyzed by one way ANOVA followed by tuckey post hoc test; SO, simple ointment base; HEO, hydroalcoholic extract ointment; NFO, nitrofurazone ointment;.numbers (3-15) refer days where measurment was taken following wound creation; 0 values refer day where complete healing was achieved; ^a^against control, ^c^against 5 % (w/w) hydroalcoholic extract; ^2^ *p* < 0.01, ^3^ *p* < 0.001

#### Epithelization period

Table 3 shows epithelization period of excision wound of all formulations tested. Animals treated with the extract as well as the standard exhibited shorter epitthelization period than the controls. Time for epithelization was shorter by 23.1 % for 10 % (*p* < 0.001), 22.1 % for nitrofurazone (*p* < 0.001) and 16.3 % for 5 % (*p* < 0.05) compared to those treated with the ointment base. However, no apparent difference in epithelization period was found between the extract and the standard as well as between the different concentrations of the extract.

**Table 3:** Effect of topical application of the ointment formulated from 80 % methanol extract of the rhizomes of *Rumex abyssinicus* on epithelization period

Group	Epithelization period (days)
SO	17.3 ± 0.333
5 % HEO	14.5 ± 0.764^a2^
10 % HEO	13.3 ± 0.333^a3^
NFO	13.5 ± 0.671^a3^

*n* = 6 animals in each group; Values are expressed as mean ± SEM and analyzed by one way ANOVA followed by tuckey post hoc test; SO, simple ointment base; HEO, hydroalcoholic extract ointment; NFO, nitrofurazone ointment;. ^a^against control; ^2^ *p* < 0.01, ^3^ *p* < 0.001

#### Estimation of hydroxyproline content

Hydroxyproline content of wound tissues following 10 days after treatment is depicted in Table 4. Hydroxyproline levels were significantly greater in animals treated with the extract as well as the standard compared to controls. Looking at the percentage increase, nitrofurazone achieved the maximum increase (102.2 %, *p* < 0.001) followed by 10 % (74.2 %, *p* < 0.001) and 5 % (31.6 %, *p* < 0.05) of the extract ointment. Once again, although there appeared to be a difference in mean increase values among the treatment, the difference did not reach statistical significance.

**Table 4:** Hydroxyproline content of excision wounds following topical application of the ointment formulated from 80 % methanol extract of the rhizomes of *Rumex abyssinicus*

Group	Hydroxyproline (mg/100 g)
SO	10.6 ± 0.92
5 % HEO	13.9 ± 0.89^a1^
10 % HEO	18.5 ± 0.70^a3^
NFO	21.6 ± 0.86^a3^

*n* = 6 animals in each group; Values are expressed as mean ± SEM and analyzed by one way ANOVA followed by tuckey post hoc test; Hydroxyproline was determined following treatment for 10 days; SO, simple ointment base; HEO, hydroalcoholic extract ointment; NFO, nitrofurazone ointment;. ^a^against control;^1^ *p* < 0.05, ^3^ *p* < 0.001

### Incision wounds

The mean tensile strength in the group treated with simple ointment base BP tended to increase by about 19.4 % compared to untreated controls, which failed to reach statistical significance. Tensile strength was, however, significantly increased by about 36.2 % (*p* < 0.05), 57.2 % (*p* < 0.001), and 79.1 % (*p* < 0.001) with 5 %, 10 % of the extract and nitrofurazone ointment, respectively, compared to controls treated with the ointment base (Table 5). The tensile strength of animals treated with the standard drug was significantly higher (*p* < 0.01) than the 5 % extract treated animals, although no apparent difference was detected with 10 % formulation of the extract.

**Table 5:** Effect of topical application of the ointment formulated from 80 % methanol extract of the rhizomes of *Rumex abyssinicus* on tensile strength of incision wound

Group	Tensile strength (g) (mean ± SEM)	% Tensile strength
Untreated control	205.4 ± 19.5	-
SO	244.67 ± 17.1	19.4 %
5 % HEO	333.33 ± 18.2^a1^	36.2 %
10 % HEO	384.50 ± 19.6^a3^	57.2 %
NFO	438.17 ± 19.1^c2,a3^	79.1 %

*n* = 6 animals in each group; Values are expressed as mean ± SEM and analyzed by one way ANOVA followed by tuckey post hoc test; tensile strength was measured on the 10^th^ post-wounding day using continuous water flow technique; SO, simple ointment base; HEO, hydroalcoholic extract ointment; NFO, nitrofurazone ointment; ^a^against control treated with simple ointment base;c against 5% (w/w) hydroalcoholic extract;^1^ *p* < 0.05, ^2^ *p* < 0.01, ^3^ *p* < 0.001

### Anti-inflammatory test

Table 6 summarizes anti-inflammatory activity of the extract in mouse paw edema. Treatment with neither the extract nor the standard produced anti-inflammatory activity one hour following inflammation induction compared to controls. However, the two higher doses of the extract (500 and 750 mg/kg) as well as the standard drug significantly (*p* < 0.001) inhibited edema after 2 h of carrageenan injection. On the other hand, the lower dose (250 mg/kg) of the extract displayed anti-inflammatory activity (*p <* 0.05) after 3 h of edema induction.

**Table 6:** Anti-inflammatory activity of 80 % methanol extract of the rhizomes *of Rumex abyssinicus* on carrageenan-induced mice paw edema following oral administration

Group	Mean change in the paw volume (ml)				
	Basal	1 h	2 h	3 h	4 h
Control	0.50 ± 0.04	0.61 ± 0.02	0.7 ± 0.03	0.69 ± 0.04	0.65 ± 0.03
Extract (250 mg/kg)	0.50 ± 0.02	0.60 ± 0.03 (1.64 %)	0.58 ± 0.04 (17 %)	0.55 ± 0.02^a1^ (20.29 %)	0.51 ± 0.02^a2^ (21.54 %)
Extract (500 mg/kg)	0.50 ± 0.03	0.55 ± 0.02 (9.84 %)	0.47 ± 0.04^a3^ (32.86 %)	0.44 ± 0.02^a3^ (36.23 %)	0.30 ± 0.03^a3c3^ (53.85 %)
Extract (750 mg/kg)	0.51 ± 0.01	0.48 ± 0.04 (21.31 %)	0.43 ± 0.02^a3,c1^ (38.57 %)	0.40 ± 0.04^a3,c2^ (42.03 %)	0.25 ± 0.02^a3c3^ (61.54 %)
Indomethacin (10 mg/kg)	0.53 ± 0.03	0.48 ± 0.03 (24.6 %)	0.35 ± .02^a3c3^ (50 %)	0.24 ± 0.02^a3c3^ (65 %)	0.10 ± 0.02^a3c3^ (84.6 %)

*n* = 6 animals in each group; Values are expressed as mean ± SEM, with inhibition deppicted in parenthesis; data were analyzed by one way ANOVA followed by tuckey post hoc test;; ^a^against control, ^c^against 250 mg/kg; ^1^ *p <* 0.05*,* ^2^ *p <* 0.01*,* ^3^ *p <* 0.001

## Discussion

Wound healing is a complex process that cannot be understood by using a single model or relying on an *in vitro* study. Better understanding of the healing process necessitates the use of two or more different *in vivo* models [36]. Wound healing includes an acute inflammatory phase accompanied by collagen synthesis, which finally form a scar. Currently available drugs can’t affect all phases of wound healing, calling for the need for developing new drugs from different sources. Rapid healing requires fast wound contraction, shorter epithelization period and adequate gain of tensile strength. Biochemical markers, including tissue DNA, RNA, total protein, and hydroxyproline are indicative of better healing quality of drugs [37, 38]. Thus, in the present study, an attempt was made to evaluate the potential wound healing activity of *R. abyssinicus*, a medicinal plant used for wound healing in Ethiopian folklore medicine using the two most important wound models i.e., excision and incision wound models.

In excision wound model, the ointment formulated from the hydroalcoholic extract of the rhizomes of *R. abyssinicus* showed significant increase in percentage closure of excision wounds (Table 2 & Fig. 4). During the proliferative phase of wound healing, wound contraction enhances closure of the defect by pulling the edges of the wound towards the center [39]. Contraction decreases healing time because it decreases the size of the wound and reduces the amount of extracellular matrix needed to repair the defect. Contraction also facilitates re-epithelization by shortening the distance migrating keratinocytes must travel [40]. Moreover, the wound will close at fast rate, if the medication is more efficient [41]. The effect of the rhizomes of *R. abyssinicus* on wound contraction points to the fact that the extract is endowed with pro-healing action as wound contraction accounts for 88 % of the healing process (the remaining healing is due to scar formation) [42]. Although the mechanism by which the extract increased wound contraction remains to be seen, it is plausible to suggest that it might be attributed to either the plant’s anti-inflammatory effect (present study) or induction of macrophage cell proliferation [21].

In this study, the epithelization time was also found to be significantly shorter in animals treated with ointments containing the crude extract (Table 3). Epithelization involves the proliferation and migration of epithelial cells across the wound bed [43]. Therefore, the shorter epithelization time in the hydroalcoholic extract might be due to facilitated proliferation of epithelial cells and/or increasing the viability of epithelial cells [30]. A large body of evidence indicates that antimicrobial activity correlates with wound healing. Infection can seriously delay healing process by causing poor quality granulation tissue formation, reduced tensile strength of connective tissue, impaired epithelization and odor [14, 44]. Hence, increased rate of wound contraction and decrease in period of epithelization in the animals treated with the extract in excision wound model could also be attributed to the *R. abyssinicus’s* antimicrobial activity, as such activity is reported earlier for the plant [21]. Wound re-epithelization is a hallmark of successful wound care [45]. Moreover, better healing activity is distinguished with short epithelization period [46]. Thus, shorter epithelializtion periods in animals treated with the extract reinforces the notion that *R. abyssinicus* has a potential application as a wound healing agent.

Hydroxyproline is an amino acid that is found in collagen and measuring its level indicates collagen turnover [47]. The hydroxyproline content in mice treated with ointment containing the extract was significantly higher than controls, but comparable to the standard (Table 4). Higher hydroxyproline content in the extract and standard treated group might be related to enhancement of the proliferation and migration of fibroblasts and collagen deposition [48]. Thus, it is conceivable to suggest that the observed decrease in epithelization and increase in wound contraction with application of the extract could be due to the plant’s potential to increase collagen synthesis through its effect on hydroxyproline content, as wound contraction begins almost concurrently with collagen synthesis [1].

Better efficacy of the crude extract in wound healing was further evidenced by the breaking strength in incision wounds (Table 5). Higher tensile strength is an indication of better wound healing [49] and it mainly depends on the increase in collagen concentration and stabilization of the fibers. Hence, based on the amount of hydroxyproline detected, it could be assumed that the crude extract enhanced the strength of the incision wound by increasing the collagen levels, which could stick the wound edges together at the repaired site [45].

Inflammation is important in wound healing. During acute wound it makes wound bed to be healed by removing necrotic tissue, debris, and bacterial contaminants as well as recruiting and activating fibroblasts. Under normal conditions, inflammation is a self-limiting process. Excessive inflammation, however, limits wound healing [11]. The extract showed anti-inflammatory activity in carrageenan induced paw edema model in mice (Table 6). Carrageenan is widely used to induce hind paw edema for the discovery and evaluation of anti-inflammatory drugs [34]. Carrageenan injection induces inflammation within short period of time. It produces three distinct phases: first (0–1.5 h), second (1.5–2.5 h) and third (2.5–5 h) phases of inflammation. The mediators of inflammation involved are histamine and serotonin, bradykinin, and prostaglandins for the three phases, respectively. The third phase is mostly used to assess anti-inflammatory effect of traditional medicinal plants, as this phase is sensitive to most clinically effective anti-inflammatory drugs [50]. Neither of the treatments produced effect in the first hour, suggesting less responsiveness of phase one mediators to the tested agents (Table 5). Inhibition was, however, apparent following the 2^nd^ hour, the only exception being 250 mg/kg of the extract. This observation could possibly indicate that phase 2 mediators’ response might vary with dose. On the other hand, all doses of the extract were effective from the 3^rd^ hour onwards, regardless of the dose levels used, pointing to the fact that phase 3 mediators are the most likely targets for the extract. There appears also to be an *in vitro* and *in vivo* correlation, as *in vitro* anti-inflammatory activity for the plant has been described in previous reports [21]. Moreover, this finding is concordant with earlier reports showing that plants having anti-inflammatory activity do also exhibit wound healing effect [11].

The 80 % methanol extract of the rhizomes of *R. abyssinicus* had been reported to possess secondary metabolites, including, among others, tannins, saponins, flavonoids, steroids and anthraquinones [22]. Flavonoids and tannins have been shown to be important for wound healing due to their antioxidant, anti-inflammatory and antibacterial activities. In line with this notion, published reports indicate that hydroalcoholic extract of *Rumex crispus* [51], *R. maderensis* [52], *R. acetosa*, *R. patientia* [53], *Polygonum convolvulus*, *Rheum undulatum*, *and R. acetosella* [54] possess wound healing activity because of their phenolic (such as flavonoids and tannins) content. Active constituents such as chrysophanol, emodin and physcion were also previously isolated from the rhizomes of *R. abyssinicus* [55]. Emodin [37, 56] and chrysophanol [57] isolated from other plant species are reported to have antimicrobial, anti-inflammatory and wound healing activities. In addition, majority of the Rumex species have also been reported to have antimicrobial action due to the presence of physcion and rumicin [19]. Taken together, these findings collectively indicate that phytoconstituents individually or synergistically may initiate a host of mechanisms responsible for the wound healing activity of *R. abyssinicus.* Although histopathological studies are not performed that could have provided data with regeneration of the epithelium and disposal of the collagen, the measured parameters consistently showed that the plant is endowed with favorable wound healing activities. Preparations are underway to isolate the compound (s) responsible for the observed activities in subsequent studies.

## Conclusions

The data generated from the present study indicated that the crude extract of the rhizomes *R. abyssinicus* increased wound contraction, breaking strength of the repaired tissue and hydroxylproline content. These findings highlight the potential wound healing activity of the plant and uphold the traditional claims of the plant for the treatment of wounds. The hydroalcoholic extract was also endowed with significant anti-inflammatory activities that explain, at least in part, its wound healing activity.
